# When Illegitimate Tasks Threaten Patient Safety Culture: A Cross-Sectional Survey in a Tertiary Hospital

**DOI:** 10.3389/ijph.2023.1606078

**Published:** 2023-09-07

**Authors:** Stéphane Cullati, Norbert K. Semmer, Franziska Tschan, Gaëlle Choupay, Pierre Chopard, Delphine S. Courvoisier

**Affiliations:** ^1^ Quality of Care Service, University Hospitals of Geneva, Geneva, Switzerland; ^2^ Faculty of Medicine, University of Geneva, Geneva, Switzerland; ^3^ Population Health Laboratory (#PopHealthLab), University of Fribourg, Fribourg, Switzerland; ^4^ Department of Psychology, University of Bern, Bern, Switzerland; ^5^ National Center for Competence in Research on Affective Sciences, Geneva, Switzerland; ^6^ Biological Work and Health Psychology, University of Konstanz, Konstanz, Germany; ^7^ Institute for Work and Organizational Psychology, University of Neuchâtel, Neuchâtel, Switzerland

**Keywords:** illegitimate tasks, patient safety, safety culture, patient care management, teamwork

## Abstract

**Objectives:** The current study investigates the prevalence of illegitimate tasks in a hospital setting and their association with patient safety culture outcomes, which has not been previously investigated.

**Methods:** We conducted a cross-sectional survey in a tertiary referral hospital. Patient safety culture outcomes were measured using the Hospital Survey on Patient Safety Culture questionnaire; the primary outcome measures were a low safety rating for the respondent’s unit and whether the respondent had completed one or more safety event reports in the last 12 months. Analyses were adjusted for hospital department and staff member characteristics relating to work and health.

**Results:** A total of 2,276 respondents answered the survey (participation rate: 35.0%). Overall, 26.2% of respondents perceived illegitimate tasks to occur frequently, 8.1% reported a low level of safety in their unit, and 60.3% reported having completed one or more safety event reports. In multivariable analyses, perception of a higher frequency of illegitimate tasks was associated with a higher risk of reporting a low safety rating and with a higher chance of having completed event reports.

**Conclusion:** The prevalence of perceived illegitimate tasks was rather high. A programme aiming to reduce illegitimate tasks could provide support for a causal effect of these tasks on safety culture outcomes.

## Introduction

Illegitimate tasks are defined as tasks that people think they cannot properly be expected to carry out, either because the tasks should not exist or are not needed (unnecessary tasks), or because they are not part of their specific role as employees (unreasonable tasks) [1–3]. Such tasks do not represent meaningful contributions to the fulfilment of one’s occupational role and often detract from the core tasks that define that role [3]. The problem of illegitimate tasks is widespread in the world of work, and is observable in companies in most sectors of the economy [2, 3], including healthcare [4–6]. In hospital settings, the complexity of patient care suggests that acute care hospitals may be an environment at risk for illegitimate tasks. For example, nurses use the special term “non-nursing activities” [7, 8], and many physicians complain about non-medical tasks [5, 9, 10]. However, evidence on the prevalence of illegitimate tasks in hospital settings remains scarce. Furthermore, although illegitimate tasks have been linked to health and well-being, to the best of our knowledge no study has yet investigated their association with patient safety culture.

Risks factors for poor patient safety culture have been examined in multiple healthcare settings; the findings of this research have pointed to the importance of healthcare professionals’ characteristics, including their number of years of experience in the hospital [11] or the unit [12], their type of profession [12–18], their level of seniority in their profession [18], the number of hours worked per week [17], and sociodemographic factors [16, 19, 20]. Moreover, there is a body of literature reporting on variability in patient safety culture as related to numerous hospital characteristics, including certification [21], accreditation [11], medical departments [22], hospital size [11, 17] and type [17], and level of staffing in the units [23]. However, the way in which work and tasks are distributed and organised within units or wards has not been studied in relation to patient safety culture. Poor organisation of work and tasks is a stressor for identification with one’s professional role [24] and with the organization [25] and can hamper staff performance [25–27], for instance by rendering their use of time inefficient [28, 29]. In a healthcare setting, we can assume that tasks perceived as “illegitimate” could decrease work satisfaction among staff [4, 30], demotivate staff [5], reduce satisfaction with performance [31], and reflect a lack of good management in the unit [25]. In non-healthcare settings, perception of illegitimate tasks has been found to be associated with more counterproductive work behaviour [32, 33] and with lower self-esteem [33, 34], work engagement [5], job identity [35], and supervisor-rated task performance [35]. However, at present, we do not know whether perception of illegitimate tasks among hospital staff is associated with safety culture.

The objectives of this study were (1) to describe the prevalence of illegitimate tasks among healthcare staff in a tertiary referral hospital, and (2) to assess the association between perception of illegitimate tasks and patient safety culture.

## Methods

### Design

We conducted a cross-sectional survey using a self-administered questionnaire. Specifically, the current study used data from a census of healthcare professionals in a single hospital, the primary aim of which was to monitor the safety culture of the hospital through repeated cross-sectional surveys.

### Population and Sample

The survey was conducted at the Geneva University Hospitals, Switzerland, in November/December of 2016. The Geneva University Hospitals form a 1700-bed tertiary referral hospital for the State of Geneva, an international metropolitan area of almost one million inhabitants. The inclusion criteria for the survey were (1) being a healthcare professional and (2) working with patients, either “directly” (having direct contact, at the patient bedside), or “indirectly” (work related to patient clinical care, such as laboratory, pathology, radiology, and pharmacy work).

### Procedure

The survey was first advertised on the hospital intranet and through posters in various departments and wards. All healthcare professionals at the hospital were then contacted by email with a request to complete the online survey. Email addresses were obtained from the human resources department. Three reminders were sent, 7 days apart.

### Ethical Considerations

The survey was exempted from ethical review by the Cantonal Ethics Commission of Geneva (reference number: req-2023-00049). The confidentiality and protection of data was guaranteed in accordance with the Swiss Federal Data Protection Act.

### Dependent Variables

Safety culture was measured via two variables of the French version [36] of the Hospital Survey on Patient Safety Culture (HSOPSC) questionnaire version 1 [37]: 1) the patient safety grade in the unit/service of the respondent (hereafter, “safety rating”) and 2) whether the respondent had completed one or more safety event reports in the last 12 months. The French version of the HSOPSC questionnaire version 1 consists of 43 items measuring 12 safety culture processes (each process consisting of 3–4 items) and 2 safety culture outcomes (each outcome measured with a single item). Only the safety culture outcome items were used in this study. We hypothesised that these two outcome indicators from the HSOPSC questionnaire could serve as proxies for patient safety culture. The item assessing safety rating was “Please give your work area/unit in this hospital an overall grade on patient safety,” with the following answer options: “failing,” “poor,” “acceptable,” “very good,” and “excellent.” Safety ratings were recoded as “low” (failing and poor) or “adequate” (acceptable, very good, and excellent). The item assessing the number of safety events reports was “In the past 12 months, how many patient safety events reports have you reported?”, with the answer options “no safety event reports,” “1 to 2,” “3 to 5,” “6 to 10,” “11 to 20,” and “21 or more”; responses were recoded as “no event report” or “at least 1 event report.”

### Main Independent Variable

Perception of illegitimate tasks was measured using the French version of the Bern Illegitimate Tasks Scale (BITS; full scale reported in Supplementary Table S1) [38]. This consists of two facets: unnecessary tasks (4 items, Cronbach’s alpha = 0.85 in the analytical sample; sample item: “Do you have work tasks to take care of, which keep you wondering if they make sense at all?”) and unreasonable tasks (4 items, Cronbach’s alpha = 0.83; sample item: “Do you have work tasks to take care of, which you believe should be done by someone else?”). The two subscales reflect different aspects of illegitimacy; they may therefore be analysed separately, but the common denominator of illegitimacy is well reflected in the total scale [38], which had a Cronbach’s alpha of 0.87. Principal component analysis confirmed the unidimensionality of each dimension. To obtain a measure of the frequency of self-perceived illegitimate tasks for the description of prevalence (objective 1), we carried out the following calculations. First, we dichotomised responses to each item as either 0 (= “never, very rarely”, “quite rarely”, and “sometimes”) or 1 (= “quite often” and “very often”). Subsequently, we computed the mean value of the dichotomised responses to items, both for the entire scale and for each facet (unnecessary and unreasonable tasks). Finally, we dichotomised each continuous variable into two groups: 0–0.49 (coded as 0) and 0.50–1.00 (coded as 1), where the prevalence of illegitimate tasks was equal to either 1 (meaning that respondents answered half or more of the items with the response “quite often” or “very often”) or 0 (meaning that respondents answered less than half of the items response with the response “quite often” or “very often”; in other words, the majority of respondents gave answers such as “never, very rarely,” “quite rarely,” or “sometimes”). This measure is new and is not based on previous validation studies; we interpreted it as representing the prevalence of the perception among participants that illegitimate tasks were frequent. In the analyses related to objective 2, we treated these three variables relating to illegitimate tasks as continuous variables, using the mean scores obtained on the relevant items, ranging from 1 (“never, very rarely”) to 5 (“very often”).

### Control Variables

Healthcare professionals were asked to provide the following information: duration of their work experience in the unit/service (0–10 years vs. 11 years or more), their clinical department, and their degree of satisfaction with their work. These three factors are associated with safety culture [22, 39]. Work experience was assessed with a single question: “How long have you worked in your unit or department or sector?” The response options were “less than 1 year,” “1–5 years,” “6–10 years,” “11–15 years,” “16–20 years,” and “21 years or more,” and responses were recoded as “0–10 years” or “11 years or more.” Satisfaction with work was measured with the question “How satisfied are you with your work in general?”, and response options ranged from 1 (extremely unsatisfied) to 7 (extremely satisfied) [40]. In addition, we asked healthcare professionals about their health status; this section consisted of one item assessing their feelings of burnout or emotional exhaustion[41], one assessing their self-rated general health, and one assessing their self-esteem. The emotional exhaustion item read: “I feel burned out from my work,” with the response options “never,” “a few times a year or less,” “once a month or less,” “a few times a month,” “once a week,” and “a few times a week/every day” [42]. Self-rated general health was assessed using one question (with response options “poor,” “fair,” “good,” “very good,” “excellent”) [43], and responses were recoded to improve the continuous properties of the data [44]. This self-rated health item is a frequently used item in health research as an umbrella indicator of health, capturing the main dimension of health [45]. Finally, self-esteem was a measured using the item “I have high self-esteem” [46], with responses given on a 5-point scale ranging from 1 (“not at all”) to 5 (“absolutely”). Furthermore, we controlled for several additional self-reported characteristics: number of hours worked per week (less than 10 h, 10–29 h, 30–49 h, 50–69 h, or more than 69 h); profession (nurse, nursing auxiliary, doctor, multidisciplinary health professional, medical technician, or other profession); professional status in terms of management function (none, middle management, or top management); and whether the respondent had direct contact with patients or not.

### Statistical Analyses

Mean values for the illegitimate tasks scale (and subscales) are reported with 95% confidence intervals (95% CIs). To estimate the prevalence of illegitimate tasks, we computed the proportion of healthcare professionals who answered half or more of the questions on the Bern Illegitimate Tasks Scale with “quite often” or “very often.” The prevalence by several sociodemographic and professional control variables, such as years of experience, was also computed, and the associations between these variables and prevalence of illegitimate tasks was estimated using the chi-square test for nominal variables (e.g., department) and a test for trend for continuous variables. To assess the association between perception of illegitimate tasks (continuous) and the two dichotomised variables relating to patient safety culture outcomes (safety rating in the unit/service, and having completed one or more safety event reports in the last 12 months), we used logistic regression models, adjusting for hospital department, work-related factors (experience, hours worked, profession, managerial responsibilities, satisfaction with work), health-related factors (emotional exhaustion, self-rated health), and self-esteem. Missing data on covariates were rare (<4%) and were imputed using multiple imputation with chained equations (50 samples, predictive mean matching algorithm). Health status and self-esteem factors have previously been found to be associated with patient safety culture [19, 47] and to be predicted by the presence of illegitimate tasks [1, 4, 5, 33, 48].

We ran three robustness analyses (RAs): first, treating both exposure and outcomes as dichotomous variables (RA1); second, treating both exposure and outcomes as continuous variables (RA2); and third, treating exposure as dichotomous and outcomes as continuous variables (RA3).

## Results

Of 8,332 healthcare professionals (hereafter, “professionals”) who were invited to participate, 2,917 completed the survey (35.0% participation rate). After removal of participants with missing data on the outcomes (*n* = 302) or the main independent variable (illegitimate tasks) (*n* = 431), data from 2,478 respondents remained for analysis. Among the 2,478 respondents, data on control variables were sporadically missing, from missing emotional exhaustion scores for 0.5% of respondents to missing information on department for 3.9% of respondents.

**FIGURE 1:**
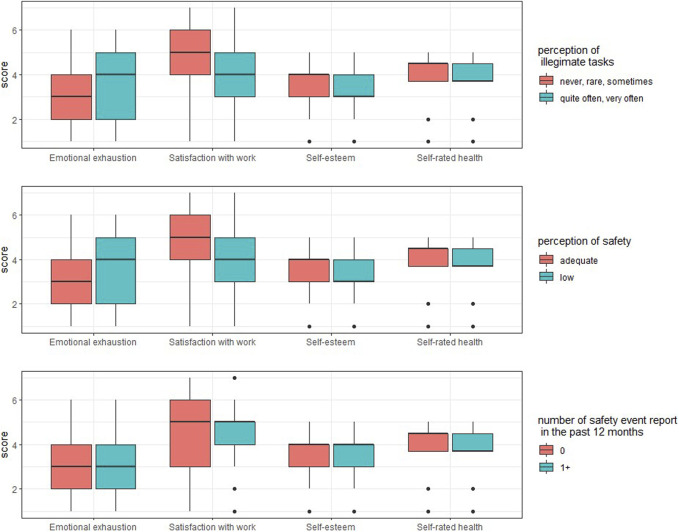
Box plot of emotional exhaustion, satisfaction with work, self-rated health and self-esteem among healthcare professionals at Geneva University Hospitals (Switzerland, 2016).

Professionals’ characteristics are reported in Table 1 and Figure 1. The professionals were mostly nurses (58.0%) and had significant amounts of professional experience, with almost two in three (65.2%) reporting 11 years or more of experience in their profession. More than three in four (77.3%) had no managerial responsibilities. Professionals reported good satisfaction with their work (mean 4.45, standard deviation (SD) 1.39, min 1, max 7) and good self-rated health (mean 4.05, SD 0.72, min 1, max 5). The average self-esteem score was 3.52 (SD 0.87, min 1, max 5). The mean level of emotional exhaustion was 3.09 (SD 1.33, min 1, max 6), and 35.6% of professionals reported feeling emotionally exhausted “every day” or “a few times a month.”

**TABLE 1:** Characteristics of a sample of healthcare professionals at Geneva University Hospitals (Switzerland, 2016).

	n (%)
Number of respondents included in the analysis	2,478
Profession	
Nurses	1,419 (58.0)
Nursing auxiliaries	251 (10.3)
Doctors	375 (15.3)
Multidisciplinary health professionals	158 (6.5)
Medical technicians	110 (4.5)
Other professions	134 (5.5)
Professional experience	
0–10 years	859 (34.8)
11 years or more	1,591 (65.2)
Managerial responsibilities	
No	1,871 (77.3)
Middle manager	373 (15.4)
Top manager	175 (7.2)
Employment rate (number of hours per week)	
Less than 29 h	297 (12.1)
30–49 h	1755 (71.7)
50–69 h	325 (13.3)
More than 69 h	72 (2.9)
Direct contact with patients at least once a week	
No	105 (4.2)
Yes	2,316 (95.8)
Hospital department	
Anaesthesiology, pharmacology, and intensive care	276 (11.6)
Surgery	234 (9.8)
Paediatrics	255 (10.7)
Gynaecology and obstetrics	119 (5.0)
Medical information sciences	74 (3.1)
Community medicine, primary care, and emergency	220 (9.2)
Genetics and laboratory	47 (2.0)
General internal medicine, rehabilitation, and geriatrics	372 (15.6)
Readaptation and palliative medicine	147 (6.2)
Psychiatry	290 (12.2)
Neurosciences	128 (5.4)
Medicine specialties	173 (7.3)
Operations	47 (2.0)
Indicators of patient safety culture (outcomes)^a^	
Patient safety rating of the unit or the department	
Excellent	116 (4.7)
Very good	1,194 (48.2)
Acceptable	969 (39.1)
Poor	157 (6.3)
Failing	42 (1.7)
Number of safety event reports in the past 12 months	
No event reports	977 (39.7)
1–2	863 (35.1)
3–5	432 (17.5)
6–10	119 (4.8)
11–20	45 (1.8)
21 or more	26 (1.1)

^a^ French version [36] of the Hospital Survey on Patient Safety Culture questionnaire version 1 [37].

Comparing the proportions of professional categories in our sample with the proportions across the whole hospital (based on the hospital’s 2016 activity reports), two groups were over-represented in our sample: nurses (58% in our sample versus 45% across the whole hospital) and multidisciplinary health professionals (7% versus 4%). For doctors, the proportion in our sample was similar to that across the hospital (15% versus 16%).

### Prevalence of Illegitimate Tasks

The response frequencies for each item of the illegitimate tasks scale (BITS) are reported in the Supplementary Table S1. Overall, 19.7% of professionals perceived illegitimate tasks to occur frequently (defined as selecting “quite often” or “very often” for half or more of their responses; hereafter, “frequent” illegitimate tasks), and 31.0% and 15.5% perceived unnecessary and unreasonable tasks, respectively, to occur frequently. The mean score on the illegitimate tasks scale was 2.62 (95% CI 2.59–2.64). The mean scores for each dimension and by profession are reported in the Supplementary Table S2.

**FIGURE 2:**
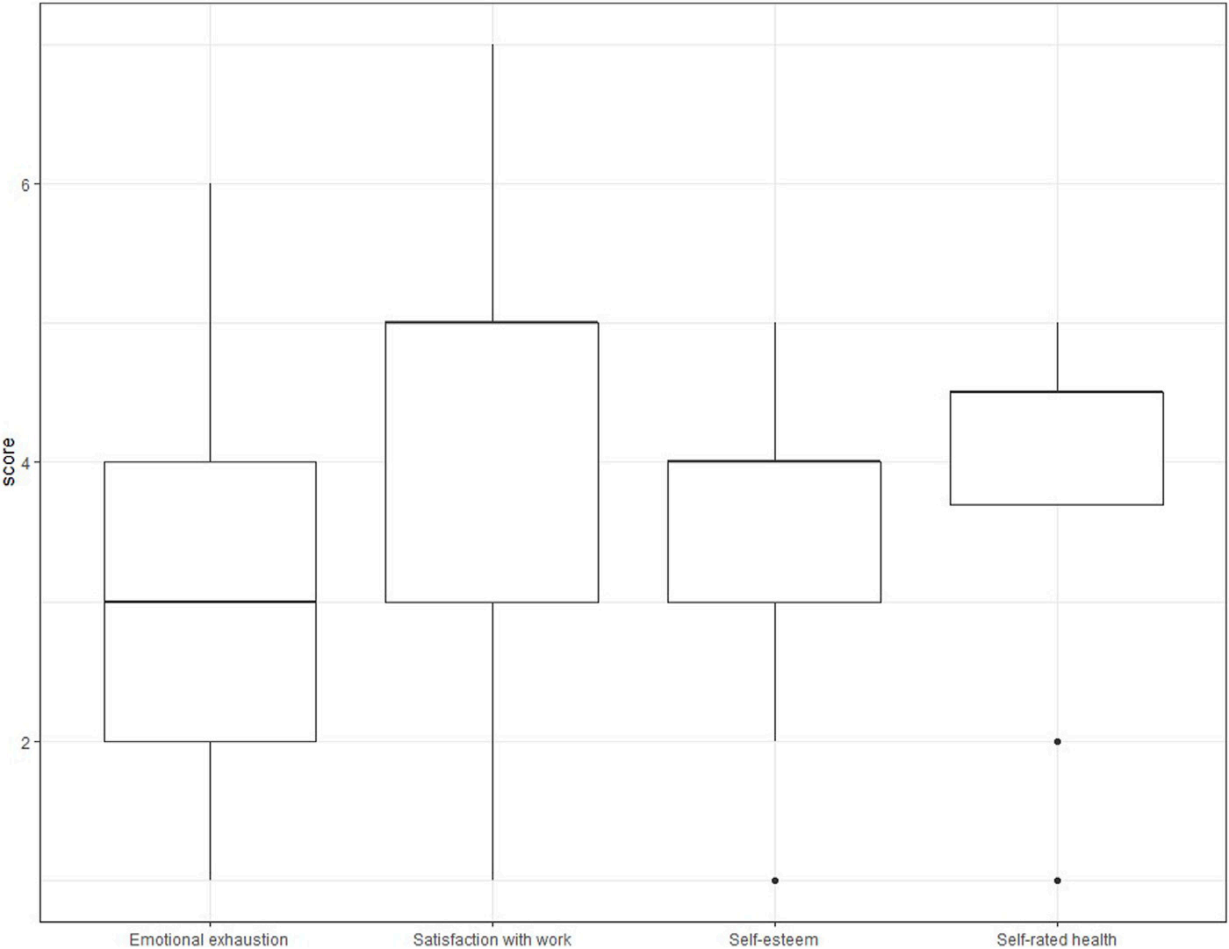
Box plots of emotional exhaustion, satisfaction with work, self-esteem, and self-rated health, by prevalence of frequent (quite/very often) illegitimate tasks (French version of the Bern illegitimate tasks questionnaire), low patient safety in the unit or service and having completed one or more safety event reports (French version of the Hospital Survey on Patient Safety Culture questionnaire version 1), (Switzerland, 2016).

The prevalence of frequent illegitimate tasks varied by profession, professional experience, managerial responsibilities, employment rate, direct patient contact, and hospital department (Table 2, first column) and by respondents’ levels of emotional exhaustion (*p* < 0.001), satisfaction with work (*p* < 0.001), self-esteem (*p* = 0.002), and self-rated health (*p* < 0.001) (Figure 2); the same patterns of variation were observed for unnecessary and unreasonable tasks (Supplementary Table S3; Supplementary Figure S1). Doctors and medical technicians, top managers, professionals with less experience (0–10 years), those working more hours per week, and those having direct contact with patients perceived illegitimate tasks, unnecessary tasks, and unreasonable tasks to occur more frequently. The perception that illegitimate tasks, unnecessary tasks, and unreasonable tasks occurred frequently was associated with greater dissatisfaction with work, lower self-esteem, poor self-rated health, and more emotional exhaustion.

**TABLE 2:** Prevalence of perceived illegitimate tasks at work and of safety culture outcomes (low patient safety rating; completion of safety event reports), overall and by professional characteristics (Switzerland, 2016).

	Reporting of frequent ^a^ illegitimate tasks^b^ (Bern illegitimate tasks scale)		Safety culture outcomes^c^			
			Reporting of low patient safety rating in the unit or service		Having completed one or more safety event reports in the past 12 months	
	n (%)		n (%)		n (%)	
Overall prevalence	487 (19.7)		199 (8.0)		1,485 (60.3)	
Prevalence by:	n (%)	*p*-value^d^	n (%)	*p*-value^d^	n (%)	*p*-value^d^
Profession		<0.001		0.113		<0.001
Nurses	248 (17.5)		128 (9.0)		1,006 (71.1)	
Nursing auxiliaries	30 (12.0)		20 (8.0)		87 (35.1)	
Doctors	125 (33.3)		23 (6.1)		211 (56.7)	
Multidisciplinary health professionals	19 (12.0)		6 (3.8)		40 (25.8)	
Medical technicians	26 (23.6)		11 (10.0)		56 (51.4)	
Other professions	34 (19.7)		8 (6.0)		67 (50.0)	
Professional experience		<0.001		0.333		0.803
0–10 years	207 (24.3)		75 (8.8)		506 (59.9)	
11 years or more	276 (17.3)		121 (7.6)		957 (60.5)	
Managerial responsibilities		0.020		0.135		<0.001
No	351 (18.8)		162 (8.7)		1,077 (57.9)	
Middle manager	77 (20.6)		23 (6.2)		263 (70.9)	
Top manager	48 (27.4)		10 (5.7)		110 (63.6)	
Employment rate (number of hours per week)		<0.001		0.285		<0.001
Less than 29 h	47 (15.8)		21 (7.1)		139 (47.1)	
30–49 h	306 (17.4)		148 (8.4)		1,088 (62.3)	
50–69 h	105 (32.3)		19 (5.8)		200 (62.1)	
More than 69 h	26 (36.1)		8 (11.1)		41 (57.7)	
Direct contact with patients		0.032		0.820		0.159
No	11 (10.9)		7 (6.9)		53 (53.0)	
Yes	465 (20.1)		187 (8.1)		1,394 (60.6)	
Hospital department		0.004		<0.001		<0.001
Anaesthesiology, pharmacology, and intensive care	34 (12.3)		6 (2.2)		208 (76.2)	
Surgery	43 (18.4)		18 (7.7)		130 (55.6)	
Paediatrics	54 (21.2)		23 (9.0)		185 (72.8)	
Gynaecology and obstetrics	32 (26.9)		6 (5.0)		58 (49.2)	
Medical information sciences	23 (31.1)		6 (8.1)		40 (54.1)	
Community medicine, primary care, and emergency	39 (17.7)		27 (12.3)		125 (57.1)	
Genetics and laboratory	5 (10.6)		4 (8.5)		20 (43.5)	
General internal medicine, rehabilitation, and geriatrics	87 (23.4)		26 (7.0)		198 (53.8)	
Readaptation and palliative medicine	25 (17.0)		11 (7.5)		84 (57.5)	
Psychiatry	57 (19.7)		41 (14.1)		175 (60.8)	
Neurosciences	31 (24.2)		9 (7.0)		76 (59.4)	
Medicine specialties	31 (17.9)		6 (3.5)		97 (56.1)	
Operations	10 (21.3)		6 (12.8)		30 (63.8)	

^a^ Frequent = half or more responses “quite often” or “very often”.

^b^ French version of the Bern Illegitimate Tasks questionnaire.

^c^ French version [36] of the Hospital Survey on Patient Safety Culture questionnaire version 1 [37].

^d^ *p*-values from chi-square tests.

### Prevalence of Safety Culture Outcomes

The proportion of professionals who reported a low safety rating (poor or failing) in their unit/service was 8.0% (Table 1). No differences in this proportion by profession, professional experience, managerial status, employment rate, or patient contact were observed, but a difference between hospital departments was observed (Table 2, second column). Professionals reported lower safety ratings when they were more dissatisfied with their work, had lower self-esteem, reported poor self-rated health, and were more often emotionally exhausted (*ps* < 0.001; Figure 2).

The proportion of professionals who reported having completed one or more event reports in the past 12 months was 60.3% (Table 1). Nurses, managers (top or middle), and professionals working more hours per week were more likely to have completed one or more event reports (Table 2, third column), as were those with fair self-rated health (*p* = 0.003) and those who were more often emotionally exhausted (*p* = 0.001); no differences according to level of work satisfaction (*p* = 0.802) or self-esteem (*p* = 0.083) were observed (Figure 2).

### Association Between Self-Reported Illegitimate Tasks and Safety Culture Outcomes

Higher perceived frequency of illegitimate tasks was associated with higher odds of a low safety rating in the unit/service (odds ratio (OR): 2.72, 95% CI 2.25–3.29; Table 3). In adjusted multivariable models, this association was attenuated, but remained significant (OR: 2.30, 95% CI 1.86–2.87). In analyses of the sub-dimensions of illegitimate tasks, higher perceived frequency of both unnecessary and unreasonable tasks was associated with higher odds of a low safety rating, in both unadjusted and adjusted models.

**TABLE 3:** Associations between perception of the frequency illegitimate tasks and two safety culture outcomes (Switzerland, 2016).

	Safety culture outcomes^a^			
	Low patient safety rating in the unit or service		Completion of one or more safety event reports	
	Unadjusted	Adjusted^b^	Unadjusted	Adjusted^b^
	OR (95% CI)	OR (95% CI)	OR (95% CI)	OR (95% CI)
Frequency of illegitimate tasks (continuous)^c^	2.72 (2.25–3.29)	2.30 (1.86–2.87)	1.34 (1.19–1.46)	1.32 (1.15–1.47)
Illegitimate tasks^c^ by dimension:				
Frequency of unnecessary tasks (continuous)	2.21 (1.86–2.63)	1.89 (1.57–2.30)	1.22 (1.10–1.32)	1.23 (1.10–1.34)
Frequency of unreasonable tasks (continuous)	2.13 (1.82–2.49)	1.80 (1.51–2.15)	1.29 (1.16–1.40)	1.23 (1.09–1.36)

OR, odds ratio; 95% CI, 95% confidence interval.

^a^ French version [36] of the Hospital Survey on Patient Safety Culture questionnaire version 1 [37].

^b^ Adjusted for work experience, hours worked per week, profession, managerial responsibilities, hospital department, satisfaction with work, emotional exhaustion, self-rated health, and self-esteem.

^c^ French version of the Bern Illegitimate Tasks questionnaire.

Higher perceived frequency of illegitimate tasks was also associated with 34% higher odds of having completed one or more event reports in the past 12 months (OR: 1.34, 95% CI 1.19–1.46). In adjusted multivariable models, this association was slightly attenuated, but remained significant (OR: 1.32, 95% CI 1.15–1.47). In analyses of the sub-dimensions of illegitimate tasks, higher perceived frequency of both unnecessary and unreasonable tasks was associated with higher odds of having completed event reports, in both unadjusted and adjusted models.

### Robustness Analyses

We checked the robustness of the main analysis by testing different coding schemes for the exposure and outcome variables (Supplementary Table S4). First (RA1), we treated the illegitimate tasks variable (and the sub-dimensions of this scale) as a dichotomous variable. For the safety grade outcome, the results were similar to those obtained in the main analysis. For the completed safety event reports outcome, the results were similar for illegitimate tasks overall, but the association with the perception that unreasonable tasks occurred frequently became non-significant in the adjusted model (OR: 1.30, 95% CI 0.99–1.63). Second (RA2), we treated both the outcome and the exposure variables as continuous variables. All results were similar to those obtained in the main analysis. Of note, higher perceived frequency of unreasonable tasks was marginally associated with having completed safety event reports in the adjusted model (b = 0.08, 95% CI 0.03–0.12). Third (RA3), we treated the outcome variables as continuous variables and the exposure variables as dichotomous variables. For safety grade, the results were similar to those obtained in the main analysis. For completion of safety event reports, the results were similar for illegitimate tasks overall, but higher perceived frequency of unreasonable tasks was only marginally associated with this outcome in the adjusted model (b = 0.12, 95% CI 0.04–0.27).

## Discussion

### Illegitimate Tasks

The mean score for perceived frequency of illegitimate tasks—the tasks that people feel they should not have to do—was 2.62 (95% CI 2.59–2.65) as rated on the BITS scale, with response options ranging from 1 to 5, among our sample of healthcare professionals working in a tertiary referral hospital. Despite the rather low rate of participation in our survey (35%), this result was similar to that of a study by [6], who reported a mean score of 2.66 for the perceived frequency of *unreasonable* tasks among physicians working in a Norwegian hospital (participation rate: 72%); the corresponding score in our subgroup of physicians was 2.59 (95% CI 2.48–2.67). It is worth noting, although the sample is not directly comparable with ours, that [4] reported a mean value of 3.06 for the perceived frequency of illegitimate tasks among general practitioners in Germany, in a study with an approximate participation rate of 6%. In studies carried out in Switzerland but with non-health professions, the mean values reported range between 1.46 [34] and 2.64 [49]. Thus, with regard to mean values on the BITS scale, the perceived frequency of illegitimate tasks remains within a range comparable to that reported among non-health professions.

In terms of prevalence, almost 20% of the participants perceived illegitimate tasks as occurring frequently (according to our coding scheme for the BITS scale, i.e., the mean of dichotomised items). It is worth noting that illegitimate tasks were perceived as occurring frequently by one-third of physicians, who thus reported the highest prevalence of frequent occurrence of illegitimate tasks among all hospital occupations (as well as on both dimensions of the illegitimate tasks scale). One hypothesis may explain this finding: with the development of healthcare management for quality improvement, all healthcare professionals are required to document their activities more regularly (reporting, indicators) and to adapt their activities to quality-oriented patient care protocols (clinical pathways). We know that doctors suffer from the burden of tasks that are perceived as “administrative” [50, 51]. In addition, with the development of evidence-based medicine, healthcare professionals need to adapt their patient care protocols to new recommendations for good clinical practice. All health professionals are affected by these developments, but it may be that doctors are more reluctant than other professionals to accommodate them, because these developments affect all professions in more or less the same way, which may be at odds with the social position of doctors in a hospital (where they are at the top of the social hierarchy) [52]. This reluctance may be reflected in the perception of a higher frequency of illegitimate tasks. After physicians, approximately 4 out of 10 professionals from the “medical technicians” group perceived illegitimate tasks as occurring frequently (24%). Considering the two dimensions of illegitimate tasks, our participants more perceived unnecessary tasks (tasks that should not exist or are not needed) as occurring more frequently than unreasonable tasks (tasks not part of their specific role), at rates of 31% and 16%, respectively; these results correspond to those reported by [5] with regard to physicians, but in the study by Kilponen et al., nurses perceived comparatively higher rates of unreasonable tasks (tasks outside their professional role). Exact comparisons with other studies are difficult, as the coding schemes used are quite different. For instance, [28] conducted a survey among staff members at 11 primary care centres in Sweden (participation rate: 89%) using a different coding scheme with the illegitimate tasks scale (taking the mean of the scale items and then dichotomising the value using a cut-off of 3.5). They reported that 3% of their participants perceived illegitimate tasks to occur at a rate above this score, with an 11% rate for the dimension of unnecessary tasks (primary care physicians 27%, nurses 9%) and 5% for the dimension of unreasonable tasks. Thus, there is no clear picture of prevalence in the healthcare field yet.

In summary: firstly, the occurrence of illegitimate tasks can certainly be regarded as a phenomenon that is non-negligible [5, 9], and doctors are probably the profession that perceive illegitimate tasks as occurring most frequently. Secondly, given that tertiary acute care hospitals provide complex care and accommodate high-needs patients, and given the growth of interdisciplinary care involving a variety of health professionals (nurses, doctors, physiotherapists, nutritionists, etc.) needing to communicate and coordinate effectively to provide the best care to patients [53–56], a high level of occurrence of perceived illegitimate tasks might have been expected in our hospital. However, the results suggested a reasonable and not excessively high level.

Our results also showed that the level of perception of illegitimate tasks is likely to vary between clinical departments in the hospital. We are not aware of any studies comparing illegitimate tasks between clinical departments. Further research to compare the level of occurrence in different clinical settings and to examine whether the risk factors differ between settings would be worthwhile.

### Safety Culture Outcomes

Roughly 60% of participants reported having completed at least one safety event report in the last year, and roughly 8% reported low safety ratings (poor or failing) in their unit. These findings suggest that the patient safety culture was relatively good in this hospital. On the one hand, a proportion of six out of ten healthcare professionals reporting having completed safety records reflects a good safety culture. This proportion was based on self-reported data, not actual reports, and may be biased, but was high compared to other hospitals, according to data from systematic reviews and meta-analysis [57, 58]. On the other hand, a rate of almost one in ten health professionals indicating that safety is low (poor or failing) in their unit is not negligible either, and this level was similar to or lower than the rates occurring in other European hospitals [59, 60].

Associations between both indicators of patient safety culture and the occurrence of illegitimate tasks emerged, as expected. A higher frequency of illegitimate tasks was associated with a higher risk of low reported patient safety ratings and a higher chance of completing at least one safety event report. These results held when controlling for work experience, hours worked per week, profession, managerial responsibilities, hospital department, satisfaction with work, burnout, self-rated health, and self-esteem; this is especially remarkable because some of these potential confounders (specifically, work satisfaction, burnout, self-rated health, and self-esteem) can themselves be regarded as being influenced by the occurrence of illegitimate tasks, as indicated by previous research [3]. To obtain significant results with these variables controlled suggests a unique role for illegitimate tasks, and the outcome variable of patient safety culture is undoubtedly an important one. Ultimately, the perception of illegitimate tasks can also impact the health of staff, with effects such as increased stress [38], increased feelings of resentment, exhaustion and burnout [1, 4, 5], poor sleep quality [61], and musculoskeletal pain [62].

The nature of the concept of illegitimate tasks implies that this type of stressor is often avoidable—indeed, the judgment that it should have been avoided is an important part of the appraisal as illegitimate. An obvious practical implication, therefore, is that it is important to analyse work processes with regard to illegitimate tasks; as this concept is not salient for many people, especially people who make decisions about task assignment [7, 8], some illegitimate tasks are likely to be assigned inadvertently, and sensitising people in leadership positions to this issue may already represent an important first step [3, 38]. If such assignments are unavoidable, explaining why they are unavoidable can mitigate, or even eliminate, the demeaning message entailed by the assignment of such tasks, and thus their stressful nature [63].

One implication of our (and others’) results is that it is important to sensitise leaders and managers to the concept of illegitimate tasks. This will require comprehensive interventions to optimise job design. Lean management [64, 65], participatory approaches assessing productivity [66], interventions aiming to improve social climate and avoid devaluing messages (e.g., CREW interventions [67]), and stress-oriented interventions in general [68], as well as the concept of MAGNET hospitals [69, 70], could be approaches to the optimisation of job design. However, the implementation of comprehensive approaches is challenging [65, 66, 71–73]. More small-scale interventions aiming to reduce illegitimate tasks, such as relieving physicians from administrative duties by employing scribes [74, 75], should not be neglected, although such approaches also require careful implementation [76].

Finally, although this study has indicated the relationship between perceptions of illegitimate tasks and two outcome indicators of safety culture, we consider these results to be a first step, as safety culture is a complex concept consisting of several dimensions. For example, the HSOPC questionnaire version 1 covers 12 safety culture processes, and in this study, we did not analyse the associations between illegitimate tasks and these safety culture processes. However, analysis of these associations may provide insight into the mechanisms that explain the relationship between illegitimate tasks and safety culture.

### Strengths and Limitations

The strengths of this study are its relatively large sample size, the use of validated scales for assessment of exposure as well as outcomes, and the consistency of the findings across adjustment, robustness, and sensitivity analyses while controlling for many potential confounders. Its limitations include the cross-sectional design, which prohibits causal conclusions. Furthermore, the participation rate was rather low. Low participation rates are of concern, but increasing them may have only a modest influence on a study’s conclusions [77], and our results for the physician subgroups were close to those of the Thun study, which had a reasonably high participation rate [6]. The influence of low participation rates relates mainly to prevalence, and not to the associations observed between variables [78–80], especially when analyses are adjusted for factors influencing participation rates. Additionally, the survey did not ask about the gender of the participants. In existing work, gender differences have not consistently been found; for instance, [81] reported a somewhat higher occurrence of illegitimate tasks for women, but neither [82] nor Omansky et al. [30] replicated this finding, although the latter reported a stronger association between illegitimate tasks and effort–reward imbalance [83] for men, which resulted in stronger negative indirect associations with job satisfaction and intrinsic motivation. The BITS scale captures an individual assessment of whether or not a professional task is legitimate, i.e., a subjective assessment, and this assessment may be independent of whether or not the task is legitimate for the hospital. Finally, our results are based on a hospital in a French-speaking culture; their generalisability has yet to be determined.

### Conclusion

The prevalence of the experience of illegitimate tasks among staff was not negligible: one in five perceived illegitimate tasks as occurring frequently. Perception of frequent occurrence of illegitimate tasks was associated with perception of low patient safety grades but with a higher number of safety event reports. Perception of illegitimate tasks is a modifiable factor, and quality improvement interventions in units with a high prevalence of illegitimate tasks may improve perceptions of patient safety among staff.
