# Supplementary material for: When Illegitimate Tasks Threaten Patient Safety Culture: A Cross-Sectional Survey in a Tertiary Hospital
Source: Int J Public Health. 2023 Sep 7;68:1606078. doi: 10.3389/ijph.2023.1606078 (PMC10511767; doi:10.3389/ijph.2023.1606078)

**When illegitimate tasks threaten patient safety culture: cross-sectional survey in a tertiary hospital**

Stéphane Cullati, Norbert K Semmer, Franziska Tschan, Gaëlle Choupay, Pierre Chopard, Delphine S Courvoisier

**Supplementary materials**

Table S1 Frequency distribution of the Bern Illegitimate Tasks questionnaire (Switzerland, 2016).

Table S2 Perceptions of illegitimate tasks at work among healthcare professionals at Geneva University Hospitals, total sample and by professions (Switzerland, 2016).

Table S3 Unnecessary and unreasonable tasks subscales of the Bern Illegitimate Tasks questionnaire (Switzerland, 2016).

Table S4 Robustness analyses of the associations between perceptions of illegitimate tasks (continuous vs. dichotomous) and two safety culture outcomes (dichotomised vs. continuous), (Switzerland, 2016).

Figure S1 Box plots of emotional exhaustion, satisfaction with work, self-esteem, and self-rated health, by prevalence of frequent (quite/very often) on the two dimensions of the Bern Illegitimate Tasks questionnaire: unnecessary und unreasonable tasks (Switzerland, 2016).

**Table S1 Frequency distribution of the Bern Illegitimate Tasks questionnaire (Switzerland, 2016).**

|  | Never, very rarely | Quite rarely | Sometimes | Quite often | Very often |
| --- | --- | --- | --- | --- | --- |
|  | n (%) | n (%) | n (%) | n (%) | n (%) |
| Do you have work tasks to take care of, which keep you wondering if… (unnecessary dimension) |  |  |  |  |  |
| they have to be done at all? | 402 (16.3) | 586 (23.7) | 1004 (40.6) | 363 (14.7) | 116 (4.7) |
| they make sense at all? | 292 (11.9) | 523 (21.4) | 994 (40.7) | 442 (18.1) | 194 (7.9) |
| they would not exist (or could be done with less effort), if things were organized differently? | 237 (9.7) | 423 (17.2) | 985 (40.1) | 595 (24.2) | 215 (8.8) |
| they just exist because some people simply demand it this way? | 386 (15.7) | 482 (19.6) | 827 (33.7) | 533 (21.7) | 229 (9.3) |
| Do you have work tasks to take care of, which you believe... (unreasonable dimension) |  |  |  |  |  |
| should be done by someone else? | 356 (14.5) | 483 (19.6) | 969 (39.3) | 450 (18.3) | 205 (8.3) |
| are going too far, and should not be expected from you? | 682 (27.7) | 795 (32.3) | 685 (27.8) | 211 (8.6) | 87 (3.5) |
| put you into an awkward position? | 786 (32.0) | 785 (31.9) | 640 (26.0) | 179 (7.3) | 68 (2.8) |
| are unfair for you to have to deal with? | 895 (36.5) | 742 (30.3) | 555 (22.6) | 182 (7.4) | 77 (3.1) |

**Table S2 Perceptions of illegitimate tasks at work among healthcare professionals at Geneva University Hospitals, total sample and by professions (Switzerland, 2016).**

|  | Perception of illegitimate tasks at work^1^ | | | | | | | | |
| --- | --- | --- | --- | --- | --- | --- | --- | --- | --- |
|  | Illegitimate tasks  (full scale) | | | Unnecessary tasks (subscale) | | | Unreasonable tasks  (subscale) | | |
|  | Mean (SD) | 95%CI |  | Mean (SD) | 95%CI |  | Mean (SD) | 95%CI |  |
| Total sample | 2.62 (0.79) | 2.59-2.64 |  | 2.88 (0.91) | 2.84-2.91 |  | 2.35 (0.88) | 2.32-2.39 |  |
|  | Mean (SD) | 95%CI | p-value^2^ | Mean (SD) | 95%CI | p-value^2^ | Mean (SD) | 95%CI | p-value^2^ |
| Profession |  |  | *<0.001* |  |  | *<0.001* |  |  | *<0.001* |
| Nurses | 2.60 (0.75) | 2.56-2.64 |  | 2.83 (0.87) | 2.79-2.88 |  | 2.36 (0.83) | 2.32-2.40 |  |
| Nursing auxiliaries | 2.42 (0.81) | 2.32-2.52 |  | 2.65 (0.89) | 2.54-2.77 |  | 2.17 (0.97) | 2.05-2.30 |  |
| Doctors | 2.88 (0.83) | 2.80-2.96 |  | 3.17 (0.96) | 3.07-3.27 |  | 2.59 (0.90) | 2.50-2.68 |  |
| Multidisciplinary health professionals | 2.41 (0.79) | 2.29-2.53 |  | 2.72 (0.91) | 2.57-2.86 |  | 2.11 (0.87) | 1.97-2.25 |  |
| Medical technicians | 2.71 (0.91) | 2.54-2.88 |  | 3.02 (1.06) | 2.82-3.22 |  | 2.41 (1.02) | 2.21-2.60 |  |
| Other professions | 2.62 (0.75) | 2.50-2.75 |  | 2.96 (0.90) | 2.81-3.12 |  | 2.28 (0.84) | 2.14-2.43 |  |

*Notes*: SD= standard deviation; 95%CI= 95% confidence interval

^1^ French version of the Bern Illegitimate Tasks questionnaire

^2^ p-values from one-way anova.

**Table S3 Unnecessary and unreasonable tasks subscales of the Bern Illegitimate Tasks questionnaire** **(Switzerland, 2016).**

|  | Bern Illegitimate Tasks questionnaire^1^ | | | |
| --- | --- | --- | --- | --- |
|  | Reporting frequent unnecessary tasks^2^ (subscale) | | Reporting frequent unreasonable tasks^2^  (subscale) | |
|  | n(%) | | n(%) | |
| Overall | 776(31.3) | | 385(15.6) | |
|  | n(%) | p-value^3^ | n(%) | p-value^3^ |
| Profession |  | <0.001 |  | <0.001 |
| Nurses | 415 (29.3) |  | 196 (13.8) |  |
| Nursing auxiliaries | 60 (23.9) |  | 32 (12.9) |  |
| Doctors | 164 (43.7) |  | 99 (26.4) |  |
| Multidisciplinary health professionals | 37 (23.6) |  | 15 (9.5) |  |
| Medical technicians | 45 (40.9) |  | 19 (17.3) |  |
| Other professions | 44 (32.8) |  | 21 (15.7) |  |
| Professional experience |  | 0.016 |  | 0.005 |
| 0 to 10 years | 294(34.5) |  | 158(18.6) |  |
| 11 years and more | 472 (29.7) |  | 225 (14.2) |  |
| Managerial responsibilities |  | 0.015 |  | 0.039 |
| No | 574 (30.7) |  | 271 (14.5) |  |
| Middle manager | 113 (30.3) |  | 70 (18.8) |  |
| Top manager | 72 (41.1) |  | 34 (19.4) |  |
| Employment rate (number of hours per week) |  | <0.001 |  | <0.001 |
| Less than 29 hours | 81 (27.4) |  | 34 (11.4) |  |
| 30 to 49 hours | 508 (29.0) |  | 243 (13.9) |  |
| 50 to 69 hours | 146 (44.9) |  | 83 (25.5) |  |
| More than 69 hours | 33 (45.8) |  | 23 (31.9) |  |
| Direct contact with patients |  | 0.371 |  | 0.086 |
| No | 27 (26.7) |  | 9 (9.0) |  |
| Yes | 728 (31.5) |  | 367 (15.9) |  |
| Hospital Departments |  | 0.017 |  | 0.006 |
| Anaesthesiology, pharmacology and intensive care | 66 (24.0) |  | 23 (8.4) |  |
| Surgery | 71 (30.3) |  | 35 (15.0) |  |
| Paediatrics | 82 (32.2) |  | 52 (20.5) |  |
| Gynaecology and obstetrics | 43 (36.1) |  | 19 (16.0) |  |
| Medical information sciences | 36 (48.6) |  | 16 (21.6) |  |
| Community Medicine, Primary Care and Emergency | 63 (28.6) |  | 30 (13.6) |  |
| Genetics and laboratory | 14 (29.8) |  | 4 (8.5) |  |
| General Internal Medicine, Rehabilitation and Geriatrics | 127 (34.1) |  | 72 (19.4) |  |
| Readaptation and palliative medicine | 39 (26.7) |  | 26 (17.7) |  |
| Psychiatry | 95 (32.8) |  | 40 (13.8) |  |
| Neurosciences | 40 (31.3) |  | 25 (19.7) |  |
| Medicine specialties | 51 (29.5) |  | 23 (13.3) |  |
| Operations | 19 (40.4) |  | 9 (19.1) |  |

*Notes*:

^1^ French version of the Bern Illegitimate Tasks questionnaire

^2^ Frequent = half or more responses “quite often” or “very often”

^3^ p-values from Chi-square test, or Goodman and Kruskal’s gamma test, or t-test, depending on the nature of the variable.

**Table S4 Robustness analyses of the associations between perceptions of illegitimate tasks (continuous vs. dichotomous) and two safety culture outcomes (dichotomised vs. continuous), (Switzerland, 2016).**

|  | Safety culture^1^ outcomes treated as: | | | |
| --- | --- | --- | --- | --- |
|  | “dichotomised” | | | |
| **Robustness analyse 1** | Low patient safety rating in the unit or service | | Completed out one or more safety event reports | |
|  | Unadjusted | Adjusted^3^ | Unadjusted | Adjusted^3^ |
|  | OR (95%CI) | OR (95%CI) | OR (95%CI) | OR (95%CI) |
| Reporting frequent illegitimate tasks (unnecessary and unreasonable)^2^ (reference: Not) | 3.82 (2.83-5.16) | 2.97 (2.13-4.16) | 1.40 (1.13-1.72) | 1.40 (1.10-1.76) |
| *Illegitimate tasks by dimensions:* |  |  |  |  |
| Reporting frequent unnecessary tasks (reference: Not) | 3.93 (2.92-5.32) | 3.04 (1.57-2.30) | 1.34 (1.11-1.58) | 1.33 (1.08-1.60) |
| Reporting frequent unreasonable tasks (reference: Not) | 3.34 (2.43-4.57) | 2.54 (1.81-3.65) | 1.35 (1.06-1.68) | 1.30 (0.99-1.63) |
|  | “continuous” | | | |
| **Robustness analyse 2** | Lower patient safety rating in the unit or service | | Number of safety event reports | |
|  | Unadjusted | Adjusted^3^ | Unadjusted | Adjusted^3^ |
|  | b (95%CI) | b (95%CI) | b (95%CI) | b (95%CI) |
| Frequency of illegitimate tasks (continuous)^2^ | 0.32 (0.28-0.35) | 0.25 (0.21-0.29) | 0.15 (0.09-0.20) | 0.13 (0.07-0.17) |
| *Illegitimate tasks by dimensions:* |  |  |  |  |
| Frequency of unnecessary tasks (continuous) | 0.24 (0.20-0.27) | 0.18 (0.15-0.21) | 0.11 (0.06-0.15) | 0.10 (0.06-0.14) |
| Frequency of unreasonable tasks (continuous) | 0.25 (0.22-0.29) | 0.19 (0.16-0.22) | 0.12 (0.07-0.16) | 0.08 (0.03-0.12) |
| **Robustness analyse 3** | b (95%CI) | b (95%CI) | b (95%CI) | b (95%CI) |
| Reporting frequent illegitimate tasks (unnecessary and unreasonable)^2^ (ref. Not) | 0.43 (0.35-0.50) | 0.33 (0.25-0.40) | 0.17 (0.07-0.27) | 0.16 (0.06-0.26) |
| *Illegitimate tasks by dimensions:* |  |  |  |  |
| Reporting frequent unnecessary tasks (reference: Not) | 0.38 (0.32-0.44) | 0.29 (0.23-0.35) | 0.17 (0.08-0.26) | 0.16 (0.07-0.24) |
| Reporting frequent unreasonable tasks (reference: Not) | 0.39 (0.32-0.48) | 0.28 (0.21-0.36) | 0.16 (0.04-0.27) | 0.12 (0.04-0.27) |

*Note*: OR= odds ratio; b coefficients are unstandardized

^1^ French version of the Hospital Survey on Patient Safety Culture questionnaire version 1

^2^ French version of the Bern illegitimate tasks questionnaire

^3^ adjusted for work experience, hours worked per week, profession, managerial responsibilities, hospital department, satisfaction with work, emotional exhaustion, self-rated health and self-esteem.

**Figure S1 Box plots of emotional exhaustion, satisfaction with work, self-esteem, and self-rated health, by prevalence of frequent (quite/very often) on the two dimensions of the Bern Illegitimate Tasks questionnaire: unnecessary und unreasonable tasks (Switzerland, 2016).**


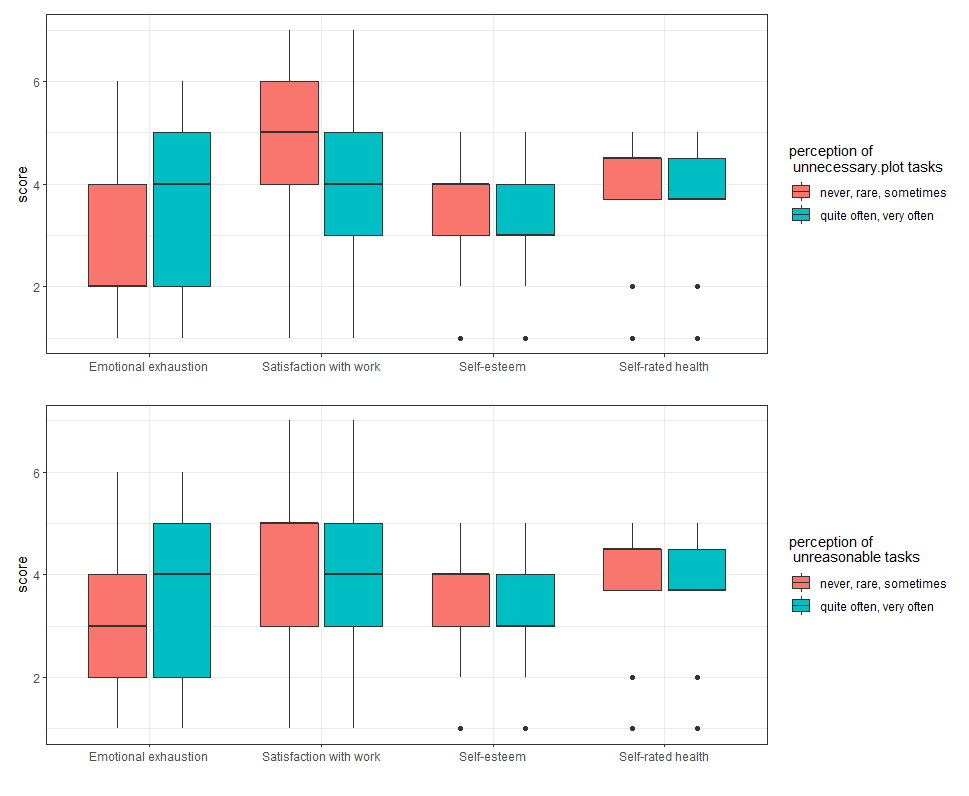

Supplement: Supplementary file 1 [file DataSheet1.docx]
